# Physical Activity and Risks of Esophageal and Gastric Cancers: A Meta-Analysis

**DOI:** 10.1371/journal.pone.0088082

**Published:** 2014-02-06

**Authors:** Yi Chen, Chaohui Yu, Youming Li

**Affiliations:** Department of Gastroenterology, the First Affiliated Hospital, College of Medicine, Zhejiang University, Hangzhou, Zhejiang Province, China; Nanjing Medical University, China

## Abstract

**Background:**

The incidence of esophageal and gastric cancer has been increasing rapidly worldwide in recent years, although the reason for this increase is unclear. Here, a statistical synthesis of studies that evaluated the association between physical activity, a well-known protecting factor against death and other chronic diseases, and the risk of esophageal and gastric cancer was performed.

**Methods:**

Potentially suitable studies were identified using Medline and Embase. The reference lists of all included articles and those of several recent reviews were searched manually. Studies were included if they (1) were published as case-control or cohort studies evaluating the association between physical activity and risk of esophageal or gastric cancer; and (2) reported point estimates (i.e., risk ratios, odds ratios) and measures of variability (i.e., 95% confidence intervals [CIs]) for physical activity and risk of esophageal or gastric cancer.

**Results:**

Fifteen studies were identified (7 cohorts, 8 case-controls; 984 esophageal and 7,087 gastric cancers). Collectively, they indicated that the risk of gastric cancer was 13% lower among the most physically active people than among the least active people (RR = 0.87, 95% confidence interval [CI] = 0.78 to 0.97) and that of esophageal cancer was 27% lower (RR = 0.73, 95% CI = 0.56 to 0.97).

**Conclusions:**

Pooled results from observational studies support a protective effect of physical activity against both esophageal and gastric cancer.

## Introduction

The incidence of esophageal and gastric cancer has been increasing rapidly worldwide in recent years, although the reason for this increase is unclear. Every year, an estimated 934,000 new cases of gastric cancer and 450,000 cases of esophageal cancer are diagnosed [1]. Gastric cancer (GC) is the fourth most common type of cancer and esophageal cancer (EC) is the sixth [1]–[3]. The mortality from these cancers is high and the response to treatments during advanced stages is poor, suggesting that an effective means of reducing mortality would be through early intervention of modifiable risk factors [4]–[6].

Physical activity (PA) is defined as movements produced by skeletal muscles that results in energy expenditure [7]. It has repeatedly been shown to be associated with reductions in the risk of any-cause mortality and reductions in major causes of death, such as cardiovascular disease and cancers [8]–[14].

The relationship between physical activity and the upper digestive tract has between widely researched and reviewed [11], [15]. Both protective role and risk factor associated with physical activity have been found, but some showing no statistical significance. No pooled analysis has yet been performed. In order to provide more reliable evidence of the relationship between physical activity and gastric and esophageal cancer, a meta-analysis of observational studies was performed with a focus on the evaluation of differences in study design, study populations and risk of bias.

## Materials and Methods

### Search Strategy and Selection Criteria

In the present work, Yi Chen and Chaohui Yu independently searched Medline and Embase (from its commencement to May 2013) with no language restrictions for studies in humans of the association between physical activity and cancers of esophagus and stomach. The core search consisted of terms related to physical activity (“exercise,” “physical activity,” “walking,” and “motor activity,”) These were combined with specific terms for cancer sites of interest (“stomach,” “gastric,” “cardia,” “esophagus,” and “esophageal”) and with descriptions of cancer (“cancer,” “neoplasm,” and “carcinoma”).

The reference lists of all included articles and those of several recent reviews were also searched [11], [15]. After eliminating duplicate studies, the titles and abstracts of all articles obtained were screened by Yi Chen and Chaohui Yu to exclude those clearly not relevant. The remaining articles were read thoroughly and those met the selection criteria were included. Differences were resolved by consulting with the third author, Youming Li.

Inclusion criteria were as follows: (1) Published as case-control or cohort study evaluating the association between physical activity and risk of esophageal or gastric cancer; (2) reported point estimates (i.e., rate ratios, odds ratios) and measures of variability (i.e., 95% confidence intervals [CIs]) for physical activity and risk of esophageal or gastric cancer.

### Data Extraction and Quality Assessment

The following information was extracted from relevant studies: first author’s name, year of publication, country in which the study was performed (nationality), study design (i.e., case-control or cohort), the sexes of the participants, cancer anatomical and histological subtypes included and risk approximations for comparisons between the highest and lowest categories of physical activities. Attention was also paid to physical activity domains and whether confounders that were controlled during the analysis (i.e., age and obesity). Extracted data were inspected for concordance by two authors Yi Chen and Chaohui Yu.

If a study did not report enough data to be included in the meta-analysis (i.e., no risk estimates and/or 95% confidence intervals), the corresponding author was contacted via email and the missing data were requested at least twice. If a study reported the effect estimates of two or more domains of physical activity but did not combine them, then the results of recreational physical activity for the primary meta-analysis were used. This was because recreational physical activity is the most commonly measured domain in observational studies of physical activity and cancer. It has been suggested that it is the main modifiable aspect of energy expenditure [12], [16].

Methodological quality was assessed using three study components that might affect the strength of the association between physical activity and the risk of gastric or esophageal cancer risk [12]: study design (i.e., population-based case-control and cohort studies were believed to have a lower risk of bias and hospital-based studies were believed to have a high risk of bias); measurement of physical activity (i.e., studies that reported that the method used to measure physical activity was valid and/or reliable or was similar to another questionnaire with known validity and/or reliability were considered to have a lower risk of bias and those did not were considered high); and the confounding effects (i.e., studies that considered/controlled/matched confounding effects such as age and obesity were considered to have a lower risk of bias and those did not were considered high). Studies that showed a lower risk of bias according to all three criteria were classified as having a low risk of bias, and the rest were classified as having a high risk of bias.

### Statistical Analysis

All analyses used the STATA statistical package with the metan and metabias commands (version 12, STATA Corporation, College Station, TX, U.S.). Summary RR estimates were calculated using either RRs (for cohort studies) or ORs (for case-control studies). For case–control studies, odds ratios with 95% CI were evaluated, and for cohort studies, risk ratios with 95% CI were evaluated. With relatively low incidence worldwide, gastric or esophagus cancer affects only a small proportion of general population. Odds ratios and risk ratios were combined in the analysis and reported as a relative risk (RR). If a study reported results for males and females separately, both risk estimates were included in the primary analysis. Heterogeneity was investigated by subgroup analysis, in which the magnitude of the combined risk estimates and the respective tests of heterogeneity, and meta-regression in each stratum to assess the independent contribution of each variable to explain heterogeneity. Publication bias was evaluated using funnel plots [17], the Begg adjusted rank correlation test [18] and Egger’s test. [19].

### Subgroup Analysis

Six pre-specified subgroup analyses were conducted, one each by sex (males vs females); by study type (cohort vs case-control); by risk of bias (lower vs higher risk of bias); by study population; and by physical activity domain (occupational, recreational).

Meta-regression analysis was used to calculate ratios of risk estimates to test for statistically significant effect modification by sex, study type, risk of bias, study population, and domain of physical activity.

## Results

### Search Results

879 potentially relevant articles were screened. Of these, 91 were considered potentially valuable and full texts were retrieved for detailed evaluation. Of these 91 articles, 79 were subsequently excluded from the meta-analysis for various reasons. Of these, 70 were excluded for not evaluating the relationship between physical activity and esophageal or gastric cancer specifically. Another 5 were then excluded because the cancer death rate was investigated, and cancer risk was not [20]–[24]. Another 3 articles because they did not provide point estimates with confidence intervals and the authors did not reply us to require for further detailed data [25]–[27]. Another study was dropped because it was cross-sectional [28]. An additional 3 articles were included from reference reviews. In this way, a total of 15 articles (7 cohort and 8 case–control studies) were included. (Figure S1).

### Study Characteristics

The main characteristics of the 15 studies included in the primary meta-analysis are given in Table 1. Of all the studies included, 7 were cohort studies [29]–[35] and 8 were case-control studies [36]–[43]. A total of 7,087 GC patients and 984 EC patients were identified among 1,507,436 participants. Six studies were conducted in Asia [two in Japan [32], [34], two in China [36], [39], one in Korea [30] and one in Turkey [43]). Three were conducted in Europe (onein multiple European countries [35], one in UK [33], and one in Norway [31]]. Four studies were conducted in the United States [29], [38], [41], [42] and two were in Canada [37], [40]. Five studies involved males only [30], [33], [37], [41], [42], eight involved both males and females but provided combined data only [29], [31], [32], [35], [39], [40], [43], [44], and only two involved both males and females and did report sex-specific results [34], [38]. All the fifteen articles provided data regarding the risk of gastric cancer, of which five did anatomical subtype analysis including cardiac gastric cancer and noncardiac [29], [31], [35], [38], [40]. One focused on cardiac cancer only [44]. Eight studies reported relationship among EC and physical activity, one [29] of which offered histological subtype analysis including esophageal squamous cell carcinoma (ESCC) and esophageal adenocarcinoma (EA) separately, one [43] on ESCC only and one [35] only about EA. The main results of four studies were based on occupational activity only [38], [41]–[43]. Five studies reported recreational activity [30], [31], [33], [40], [44]. Two studies used total physical activity [29], [34]. One study provided data about recreational and household activity separately [39]. Three studies focused on recreational and occupational activity separately [32], [35], [37].

**Table 1:** Main characteristics of the studies in the primary meta-analysis that have investigated the association between PA and GC and EC.

First author, year, subject nationality (reference)	Sex	Study setting	Cancers investigated	Physical activity domain in main result (in subgroup analyses)	Physical activity measurement mode, validity reported, and reliability reported	Confounding effect of age and obesity considered?	Main result RR or OR (95% CI)	Dose-response *P*
**Case-control studies**								
Brownson, 1991, US (43)	Male	Cancer registry	EC, GC	OCC	Job-title based, no, no	Age: yes Obesity: no	EC: 1.43 (0.66, 3.08) GC: 0.71 (0.45, 1.11)	EC: 0.18 GC: 0.13
Dosemeci, 1993, US (42)	Male	Hospital-based	GC	OCC	Job-title based, yes, no	Age: no Obesity: no	0.91 (0.54, 1.53)	0.32
Vigen, 2006, US (39)	Both, separate	Population	EC, GC (cardiac and non-cardiac)	OCC	Job-title based, yes, yes	Age: yes Obesity: yes	Female: EC: 0.35 (0.04, 3.15) GC: 0.69 (0.21, 2.25) Male: EC: 0.77 (0.54, 1.08) GC: 0.79 (0.63,1.00)	EC: 0.07 (combined) GC: cardiac: 0.97 Noncardiac: 0.67(combined)
Campbell, 2006, Canada (41)	Both, combined	Population	GC (cardiac and non-cardiac)	REC	Self-administrated, yes, yes	Age: yes Obesity: yes	0.99 (0.73, 1.34)	0.40
Chen, 2009, China (45)	Both, combined	Hospital-based	GC (cardiac)	REC	Interview, no, no	Age: yes Obesity: no	0.58 (0.16, 2.16)	NR
Wen, 2010, China (40)	Both, combined	Hospital-based	GC	REC, HH	Interview, no, no	Age: yes Obesity: yes	0.69 (0.51, 0.99)	0.04
Parent, 2011, Canada (38)	Male	Population	GC, EC	REC, OCC	Interview, no, no	Age: yes Obesity: yes	EC: 0.54 (0.30, 0.97) GC: 1.09(0.76, 1.54)	NR
Etimadi, 2011, Turkey (44)	Both, combined	Hospital-based	EC (ESCC)	OCC	Self-administrated, no, no	Age: yes Obesity: yes	0.05 (0.01, 0.26)	0.005
**Cohort**								
Serverson, 1989, Japan (33)	Both, combined	Population	GC	OCC, REC/HH	Self-administrated, yes, no	Age: yes Obesity: yes	1.45 (1.07, 1.97)	0.101 for total PA
Wannamethee, 2001, UK (34)	Male	Population	GC	REC	Self-administrated, yes, yes	Age: yes Obesity: yes	0.60 (0.14, 2.47)	0.15
Inoue, 2008, Japan (35)	Both, separate	Population	GC	Total physical activity	Self-administrated, yes, yes	Age: yes Obesity: yes	Male: 1.04(0.84, 1.29) Female: 0.63(0.42 0.94)	Men: 0.785 Women: 0.020
Sjodahl, 2008, Norway (32)	Both, combined	Population	GC(cardiac and non-cardiac)	REC	Self-administrated, no, no	Age: yes Obesity: yes	0.5(0.3, 0.9)	0.01
Huerta, 2010, Europe (36)	Both, combined	Population	EC (EA), GC (cardiac and non-cardiac, intestinal and diffuse)	OCC, REC	Self-administrated, yes, yes	Age: yes Obesity: yes	GC: 0.69 (0.50, 0.94) EC: 0.98 (0.48, 2.01)	GC: 0.006 EC: 0.951
Leitzmann, 2009, US (30)	Both, combined	Retired Persons	EC (EA and ESCC), GC(cardiac and non-cardiac)	Total physical activity	Self-administrated, no, yes	Age: yes Obesity: yes	GC: 0.84 (0.63, 1.11) EC: 0.71 (0.56, 0.91)	GC: cardiac: 0.147 Noncardiac: 0.024 EC: ESCC: 0.759 EA: 0.240
Yun, 2008, Korea (31)	Male	Population	EC, GC	REC	Self-administrated, yes, yes	Age: yes Obesity: yes	GC: 0.94 (0.86, 0.98) EC: 0.84 (0.66, 1.06)	NR

* GC = gastric cancer; EC = Esophageal cancer; ESCC = esophageal squamous cell carcinoma; EA = esophageal adenocarcinoma; OCC = occupational physical activity; REC = recreational Physical activity; NR = not reported; HH = household physical activity; OR = odds ratio; RR = relative risk;

A total of 16 sets of results were included in the primary analysis of GC (7 sets of results for males [30], [33], [34], [37], [38], [41], [42], 2 sets of results for women [34], [38], and 7 sets of results for both sexes combined [29], [31], [32], [35], [39], [40], [44] ) and 7 of EC [3 sets of results for men [30], [38], [42], 1 set of results for women [38], and 3 sets of results for both sexes combined [29], [35], [43]].

### Risk of Bias

Five of the 15 studies were neither a cohort nor a population-based case-control study [39], [41]–[44]. Nine studies reported that the method used to measure physical activity was valid and/or reliable or similar to other valid and/or reliable questionnaires [29], [30], [32]–[35], [38], . All studies except three matched on, adjusted for, or considered the confounding effects of both age and obesity [41], [42], [44]. Of the three which did not, two were adjusted for age only [42], [44]. One was not adjusted for any confounding factors [41].

Eight studies were categorized as having a lower risk of bias according to all three criteria and were so classified [31]–[35], [38], [40], [41]. Another seven studies met zero, one, or two criteria and were classified as having a higher risk of bias [29], [30], [37], [39], [42]–[44].

### Dose-Response Analyses

A total of 16 dose-response analyses (5 in men, 1 in women, and 10 in men and women combined) were conducted in the 12 studies that examined whether there was a dose-response relationship between physical activity and esophageal or gastric cancer (four for EC [29], [35], [38], [42], [43] and ten for GC [29], [31]–[35], [38]–[42]). Four analyses of physical activity and the risk of gastric cancer (one in females, and three in male and females combined) found a statistically significant (P<.05) dose-response relationship [29], [31], [34], [39]. One analyses of physical activity and the risk of esophageal cancer (in men and women combined) found a statistically significant negative dose-response relationship [43].

### Meta-analysis

#### Primary meta-analysis

The summary relative risk of the main results from the 15 studies indicated that the risk of gastric cancer was 13% lower among the most physically active people compared that among the least active people (RR = 0.87, 95% CI = 0.78 to 0.97). (Figure S2)There was moderate heterogeneity (I^2^ = 49.8%, P = .012). A similar result was found for esophageal cancer (RR = 0.73, 95% CI = 0.56 to 0.97), again with moderate heterogeneity (I^2^ = 58.4% P = .019). There was some evidence of publication bias in the primary meta-analysis. Visual inspection of the funnel plots revealed a small degree of asymmetry, due primarily to one or two studies, in both the gastric cancer and esophageal cancer results (Figure S3). Meta-regression analyses showed no variables had a significant effect (*P*>.05 in all regression analyses) on this association (Table S1).

Results of Begg’s and Egger’s tests gave no evidence of significant publication bias in the studies considered. For the association between physical activity and GC, the *P* value of Egger’s test was 0.134 for the highest versus lowest analysis. For studies on EC, the *P* value of Egger’s test was 0.328 for the highest versus lowest.

#### Subgroup analysis

No significant difference was found in any subgroup analysis with respect to study designs, sex, risk of bias, study population, PA domain and subtype. This was true of both GC and EC. However, in several subgroup analyses about EC, considerable heterogeneity was discovered with respect to study population, PA domain and histological subtype (Table 2, Figure S4).

**Table 2:** Summary of results from the primary meta-analysis and subgroup analysis.

	GC				EC			
Meta-analysis	RR(95%CI)	I^2^, %	P	Within Group ration of results (95% CI)	RR(95%CI)	I^2^, %	P	Within Group ration of results (95% CI)
Primary meta-analysis	0.87 (0.78, 0.97)	49.6	0.012	–	0.73 (0.56, 0.97)	58.4	0.018	
Subgroup analysis								
Study design								
Case-control	0.84 (0.74, 0.96)	0	0.554	Reference	0.55 (0.28, 1.10)	73.4	0.005	Reference
Cohort	0.87 (0.73, 1.04)	68.3	0.002	0.97 (0.78, 1.21)	0.78 (0.66, 0.92)	0	0.512	0.71 (0.35, 1.43)
Sex								
Male	0.91 (0.79, 1.05)	7.6	0.368	Reference	0.81 (0.64, 1.02)	26.8	0.251	Reference
Female	0.64 (0.43, 0.93)	0	0.887	1.42 (0.94, 2.13)	0.35 (0.04, 3.15)	–	–	2.31 (0.26, 20.8)
Risk of bias								
Low	0.85 (0.71, 1.02)	61.8	0.005	Reference	0.79 (0.58,1.08)	0	0.637	Reference
High	0.90 (0.80, 1.02)	14.8	0.319	0.94 (0.76, 1.17)	0.68 (0.46, 1.02)	74.8	0.003	1.16 (0.70, 1.92)
Study population								
Europe and America	0.82 (0.73, 0.92)	1.9	0.421	Reference	0.75 (0.62, 0.90)	2.3	0.402	Reference
Asia	0.93 (0.76, 1.13)	69.2	0.006	0.88 (0.70, 1.11)	–	91.3	0.001	–
PA domain								
OCC	0.79 (0.65, 0.95)	0	0.908	Reference	–	78.7	0.003	Reference
REC	0.89 (0.74, 1.06)	62.8	0.006	0.89 (0.68, 1.16)	0.80 (0.63, 1.01)	8.8	0.334	–
Subtype								
Cardiac/EA	0.78 (0.65, 0.94)	0	0.472	Reference	0.79 (0.58, 1.08)	0	0.510	Reference
Noncardiac/ESCC	0.71 (0.54, 0.94)	60.0	0.028	1.10 (0.79, 1.53)	–	92.0	0.000	–

* GC = gastric cancer; EC = Esophageal cancer; ESCC = esophageal squamous cell carcinoma; EA = esophageal adenocarcinoma; RR = relative risk.

Case-control studies and cohort studies turned out to have similar results, but negative associations between physical activity and GC were only observed in case-controls and negative associations between physical activity and EC were only observed in cohort studies. The risk ratio of physical activity and GC was found lower in women [0.91 (0.79, 1.05)] than in men [0.64 (0.43, 0.93)], but neither showed statistical significance. Studies with higher risk of bias howed lower risk ratios in EC, but higher ratios with respect to GC. However, the difference was not significant either. When investigating variation between physical activity domains, some differences were observed. Studies investigating the effects of occupational physical activity showed a slight stronger protection for both GC and EC than studies investigating the effect of recreational activity. In anatomical subtype analysis, noncardiac gastric cancer was found to have slightly stronger relationship with physical activity than the cardiac cancers. Several analyses (study population, PA domain and histological subtype analysis) of EC could not be conducted because of considerable heterogeneity. Risk estimates were not combined.

## Discussion

The results of this meta-analysis suggest that physical activity plays a protective role in both esophageal and gastric cancer. The total estimated risk from the 15 studies indicates that the risks of gastric cancer is approximately 13% lower among the most physically active people compared with the least active people and the risk of esophageal cancer was 27% lower.

There was no strong evidence that the results differed in subgroup analysis, between men and women, between studies with a higher or lower risk of bias, between differently designed studies, or between different physical activity domains. In anatomical and histological subtype analysis, cardiac cancer showed a stronger relationship with physical activity, but not statistically significant. Several analyses (study population, PA domain and histological subtype analysis) of EC could not be conducted because of considerable heterogeneity, so no combined risk estimate was obtained. This may have been because of the small number of studies were evaluated here.

The protection against cancer provided by physical activity might be mediated by insulin or adipocytokines: (1) Physical activity reduces insulin resistance and lowers fasting insulin levels. In this way, it may reduce the risk of cancer and the risk of cancer recurrence through inhibition of cell proliferation and cellular transformations [45]–[47]; (2) Physical activity and exercise decrease the concertration of inflammatory adipocytokines and increase that of anti-inflammatory adipocytokines, which are associated with lower cancer incidence and mortality [48], [49]. However, no clear mechanism regarding the protection provided by physical activity against cancers og the upper digestive tract has been proposed.

However, this meta-analysis has limitations. First, a certain degree of significance was observed in heterogeneity across all the studies included here. Overall, the heterogeneity was significantly less pronounced in cohort studies than in case-control studies with respect to GC, but the reverse was true of EC. Subgroup analyses suggested that this heterogeneity may be partly attributed to differences in methodological quality, study population, and study design. Different methods of measuring physical activity also contributed greatly to heterogeneity. Second, this meta-analysis included 15 studies, which is not enough to conduct analyses of all assumed subgroups, especially in EC. Third, in this meta-analysis, the cancer risk of the most active individuals was compared to that of the least active. The present conclusion should be refined to state that most physically active individuals have a lower risk than the most inactive ones. Fourth, another potential limitation of the present work is the residual confounding factors that were not adjusted for in the included studies. This may have affected the results.

In conclusion, a synthesis of existing studies supports the conclusion that physical activity offers some protection against esophageal and gastric cancer. This finding suggests that future research on the relationship between physical activity and gastric and esophageal cancer should focus on those aspects of the association that remain unclear, such as whether sedentary behavior and nonaerobic physical activity are associated with higher risk of cancer and, whether the intensity of physical activity affects the association between physical activity and the risk of gastric and esophageal cancer. More studies are needed to gather more information regarding the mechanism through which physical activity may protect against these cancers and whether increases in physical activity can decrease the risk of cancer.

## Supporting Information

Figure S1 **Flow diagram of systematic literature search on physical activity and the risk of esophageal or gastric cancer.**
(DOCX)

Figure S2 **Highest versus lowest meta-analysis of physical activity and the risk of esophageal or gastric cancer.** Squares represent study-specific relative risks (RR); horizontal lines represent 95% confidence intervals (CIs); diamonds represent summary relative risks.
(DOCX)

Figure S3 **Funnel plot of risk estimates from studies that investigated the associations between physical activity and the risks of gastric cancer (A) and esophageal cancer.**
(DOCX)

Figure S4 **Subgroup analysis of (A) study design, (B) risk of bias, (C) sex, (D) PA domain, (E) study population and (F) subtype of association between physical activity and gastric or esophageal cancer.** Squares represent study-specific relative risks (RR); horizontal lines represent 95% confidence intervals (CIs); diamonds represent summary relative risks.
(ZIP)

Table S1 **Results of meta-regression of included studies.** (*P* value)
(DOCX)

Checklist S1 **PRISMA 2009 Checklist.**
(DOC)
