# Supplementary material for: Physical Activity and Risks of Esophageal and Gastric Cancers: A Meta-Analysis
Source: PLoS One. 2014 Feb 6;9(2):e88082. doi: 10.1371/journal.pone.0088082 (PMC3916353; doi:10.1371/journal.pone.0088082)
Supplement: Figure S1 — Flow diagram of systematic literature search on physical activity and the risk of esophageal or gastric cancer. (DOCX) [file pone.0088082.s001.docx]

## Screening

## Eligibility

Records after duplicates removed
(n = 880 )

Records screened
(n = 880 )

## Identification

## Included

Additional records identified through other sources
(n = 3 )

Records identified through database searching
(n = 879 )

Records excluded
(n = 786 )

Full-text articles assessed for eligibility
(n = 94 )

Full-text articles excluded, with reasons
(n = 79 )

Studies included in qualitative synthesis
(n = 15 )

Studies included in quantitative synthesis (meta-analysis)
(n = 15 )

**Figure S1.** Flow diagram of systematic literature search on physical activity and the risk of esophageal or gastric cancer.
