# Supplementary material for: Physical Activity and Risks of Esophageal and Gastric Cancers: A Meta-Analysis
Source: PLoS One. 2014 Feb 6;9(2):e88082. doi: 10.1371/journal.pone.0088082 (PMC3916353; doi:10.1371/journal.pone.0088082)
Supplement: Figure S2 — Highest versus lowest meta-analysis of physical activity and the risk of esophageal or gastric cancer. Squares represent study-specific relative risks (RR); horizontal lines represent 95% confidence intervals (CIs); diamonds represent summary relative risks. (DOCX) [file pone.0088082.s002.docx]

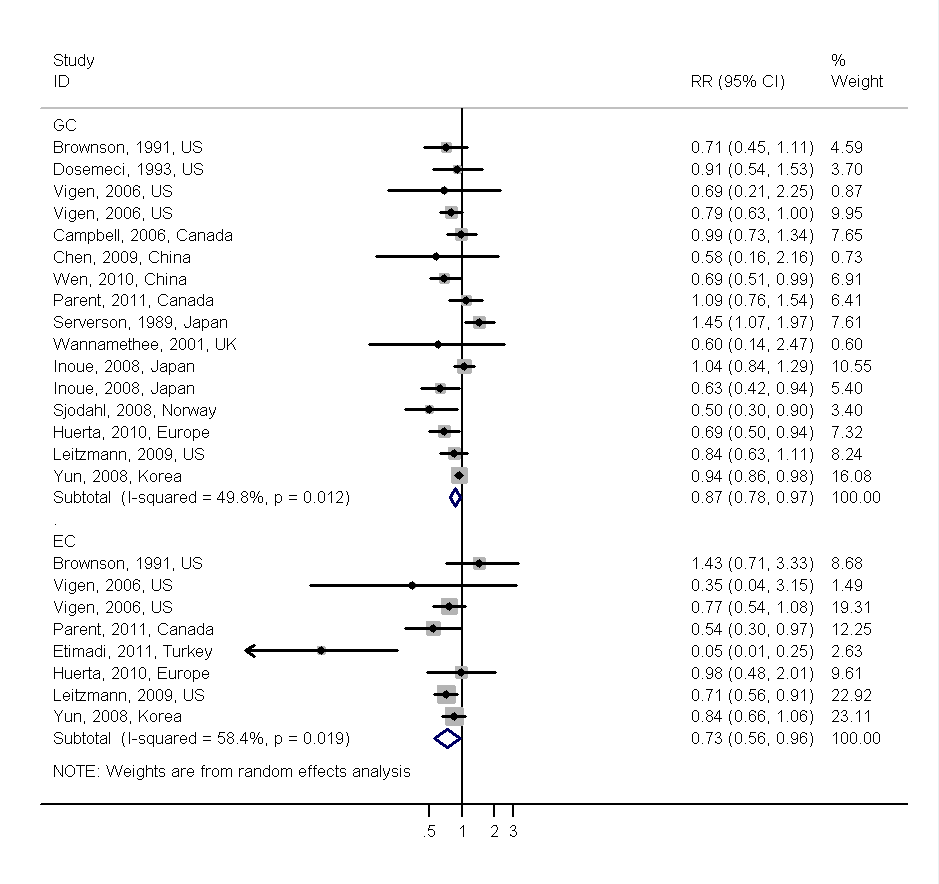


**Figure S2.** Highest versus lowest meta-analysis of physical activity and the risk of esophageal or gastric cancer. Squares represent study-speciﬁc relative risks (RR); horizontal lines represent 95 % conﬁdence intervals (CIs); diamonds represent summary relative risks.
