# Supplementary material for: Physical Activity and Risks of Esophageal and Gastric Cancers: A Meta-Analysis
Source: PLoS One. 2014 Feb 6;9(2):e88082. doi: 10.1371/journal.pone.0088082 (PMC3916353; doi:10.1371/journal.pone.0088082)
Supplement: Figure S3 — Funnel plot of risk estimates from studies that investigated the associations between physical activity and the risks of gastric cancer (A) and esophageal cancer. (DOCX) [file pone.0088082.s003.docx]

A

B

**Figure S3.** Funnel plot of risk estimates from studies that investigated the associations between physical activity and the risks of gastric cancer (A) and esophageal cancer.
