# Supplementary material for: Physical Activity and Risks of Esophageal and Gastric Cancers: A Meta-Analysis
Source: PLoS One. 2014 Feb 6;9(2):e88082. doi: 10.1371/journal.pone.0088082 (PMC3916353; doi:10.1371/journal.pone.0088082)
Supplement: Figure S4 — Subgroup analysis of (A) study design, (B) risk of bias, (C) sex, (D) PA domain, (E) study population and (F) subtype of association between physical activity and gastric or esophageal cancer. Squares represent study-specific relative risks (RR); horizontal lines represent 95% confidence intervals (CIs); diamonds represent summary relative risks. (ZIP) [file pone.0088082.s004.zip › Figure S4.docx]

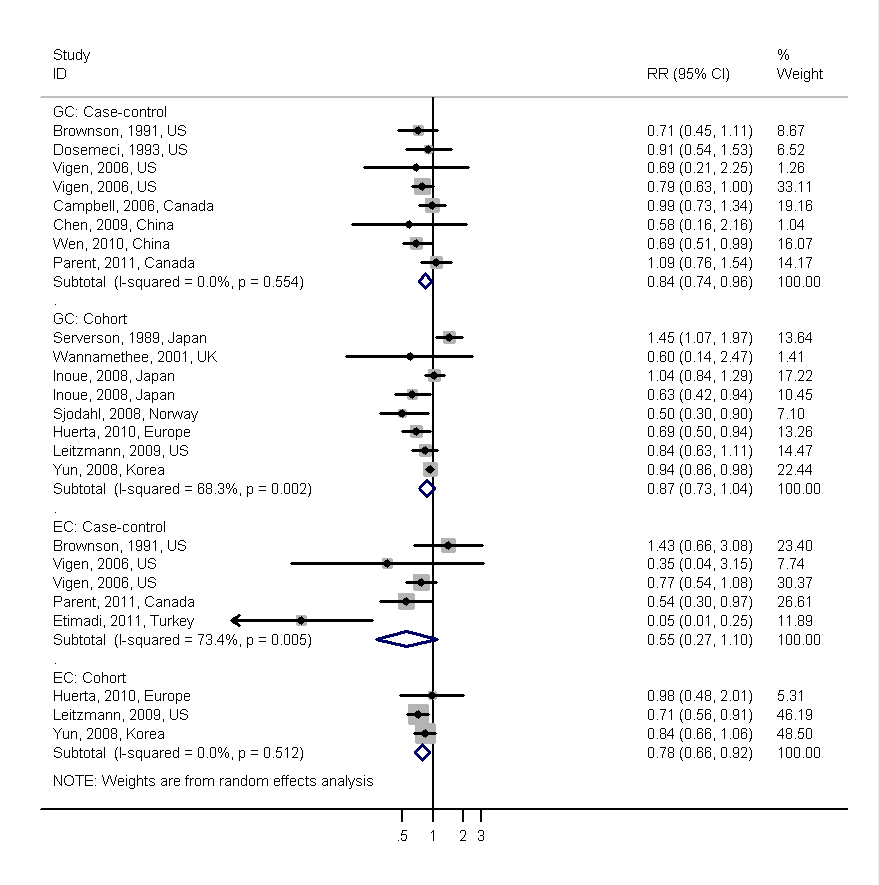

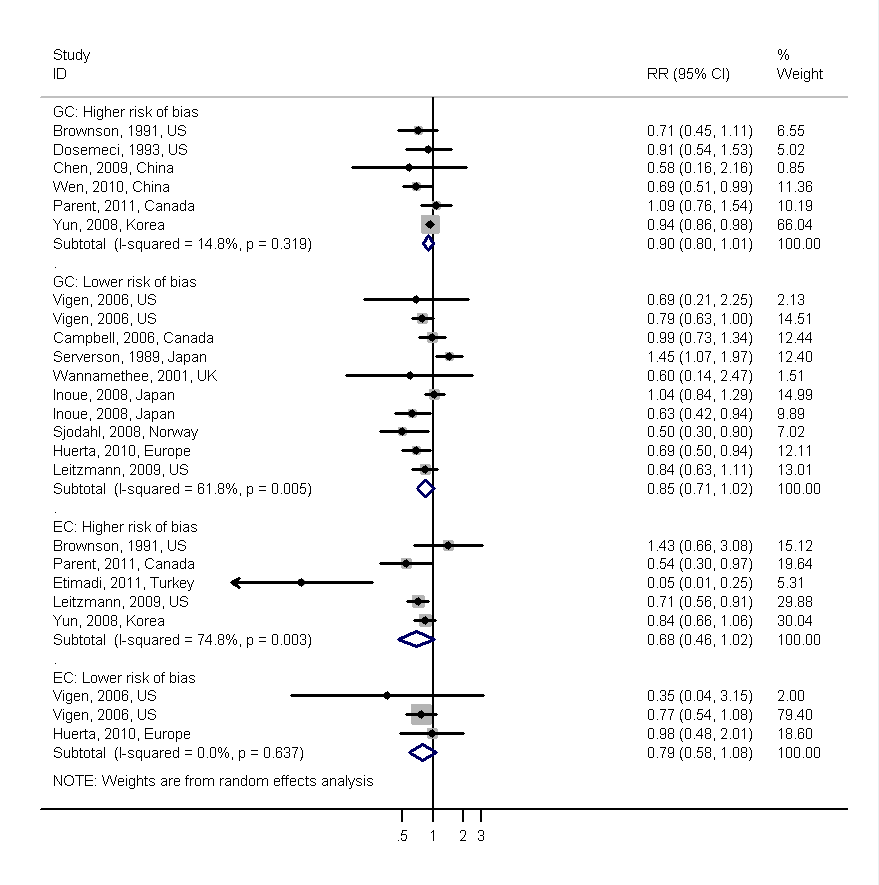


A


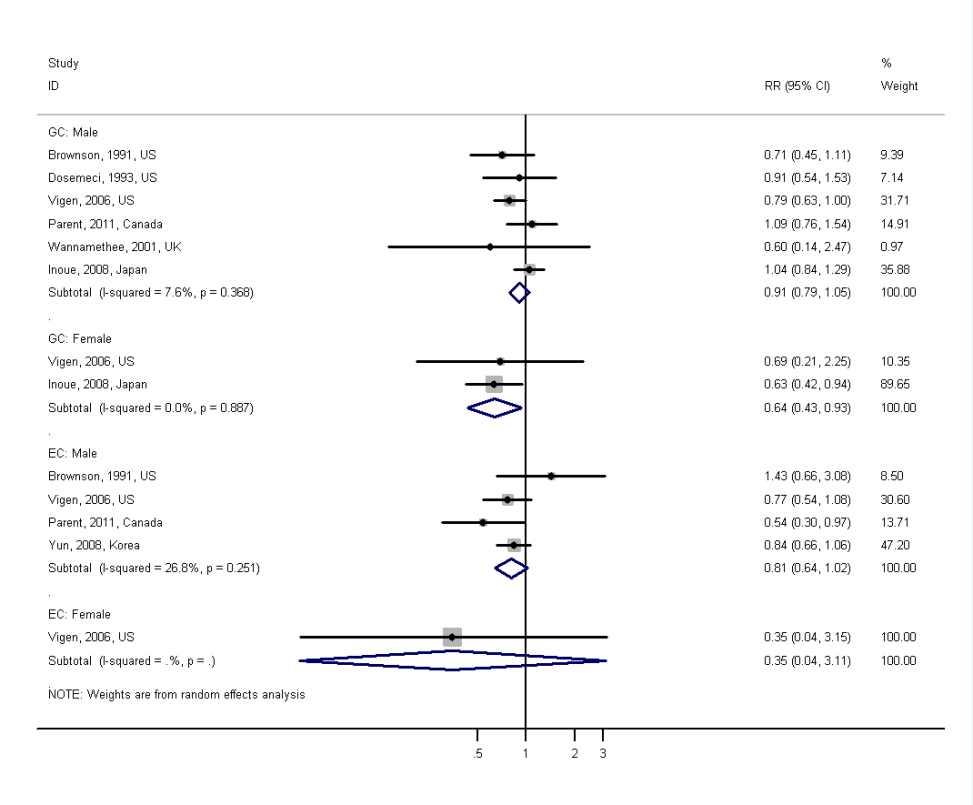

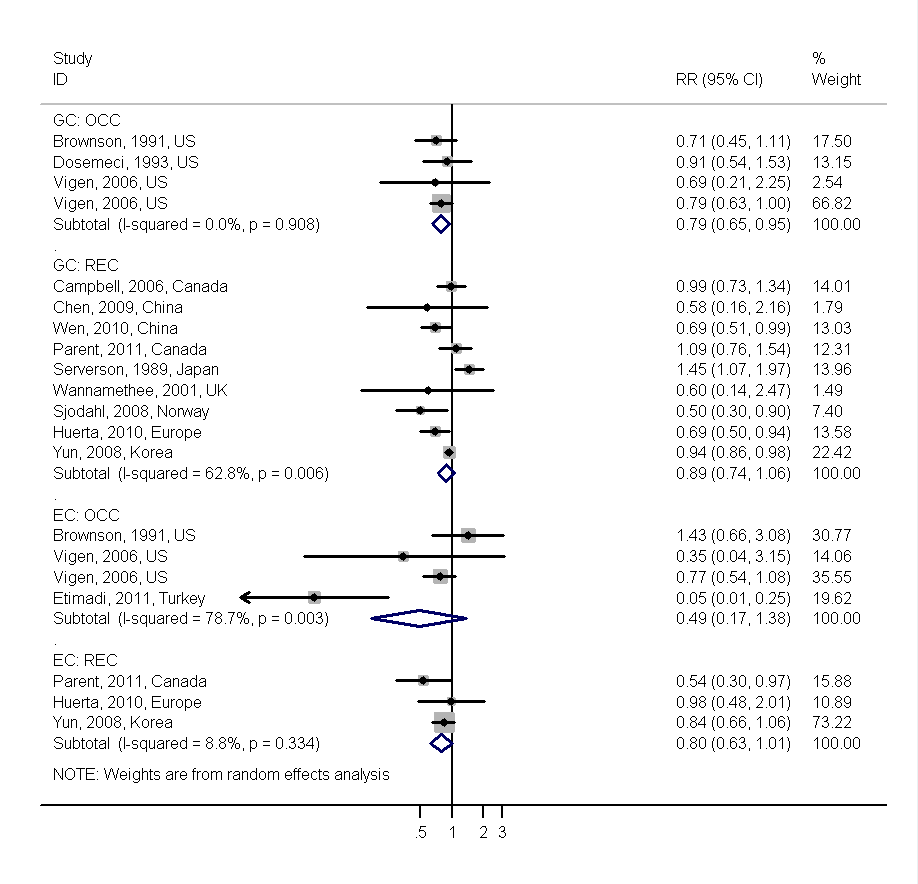


C

B


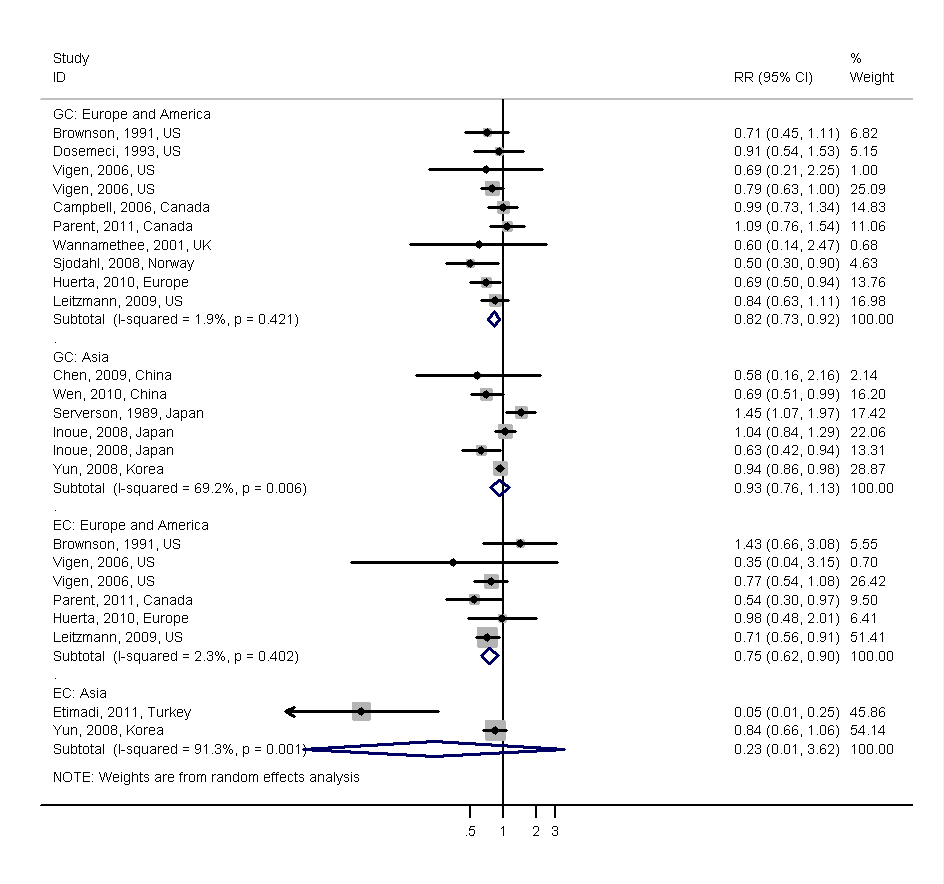

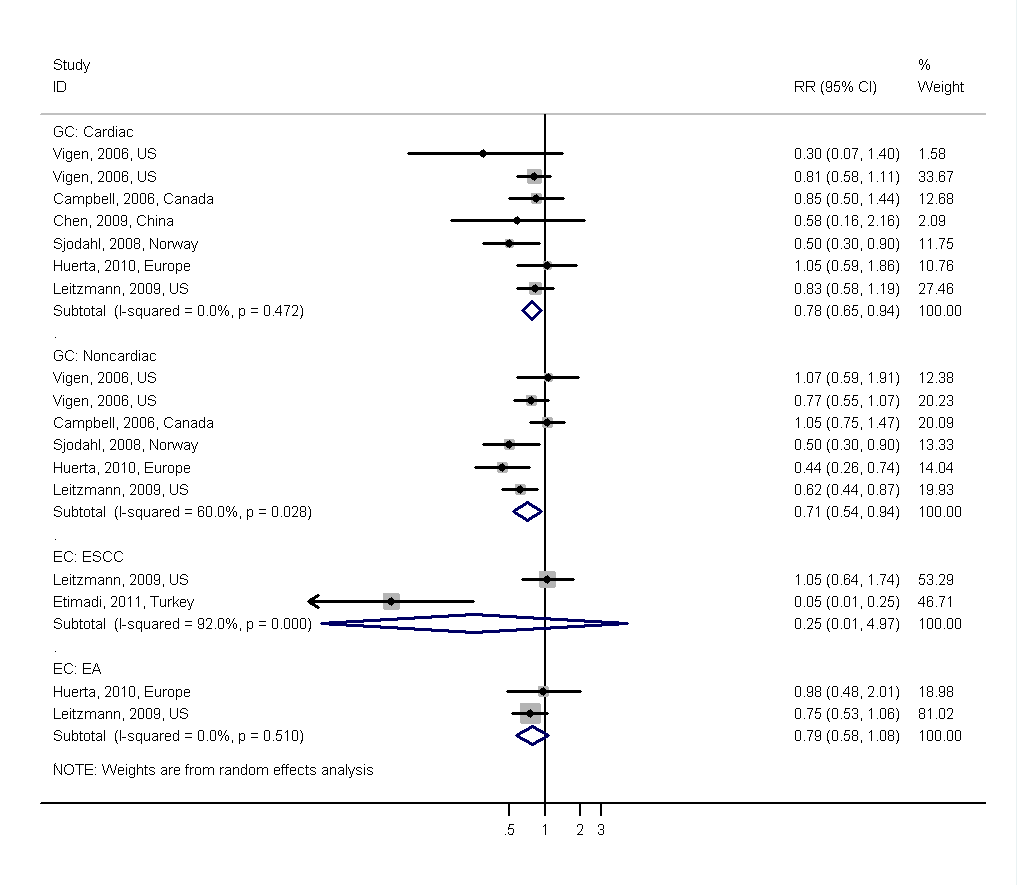


E

D

**Figure S4.** Subgroup analysis of (A) study design, (B) risk of bias, (C) sex, (D) PA domain, (E) study population and (F) subtype of association between physical activity and gastric or esophageal cancer. Squares represent study-speciﬁc relative risks (RR); horizontal lines represent 95 % conﬁdence intervals (CIs); diamonds represent summary relative risks.

F
