# Supplementary material for: Physical Activity and Risks of Esophageal and Gastric Cancers: A Meta-Analysis
Source: PLoS One. 2014 Feb 6;9(2):e88082. doi: 10.1371/journal.pone.0088082 (PMC3916353; doi:10.1371/journal.pone.0088082)
Supplement: Table S1 — Results of meta-regression of included studies. (P value) (DOCX) [file pone.0088082.s005.docx]

**Table S1.** Results of meta-regression of included studies. (*P* value)

| Variable | Study design | Sex | Risk of bias | Study population | PA domain | Subtype |
| --- | --- | --- | --- | --- | --- | --- |
| GC | 0.884 | 0.770 | 0.904 | 0.935 | 0.994 | 0.817 |
| EC | 0.177 | 0.104 | 0.486 | 0.096 | 0.171 | 0.193 |

* GC = gastric cancer; EC = Esophageal cancer; PA = physical activity
